# Park Rangers’ Behaviors and Their Effects on Tourists and Tibetan Macaques (*Macaca thibetana*) at Mt. Huangshan, China

**DOI:** 10.3390/ani4030546

**Published:** 2014-09-15

**Authors:** Rie Usui, Lori K. Sheeran, Jin-hua Li, Lixing Sun, Xi Wang, Alexander J. Pritchard, Alexander S. DuVall-Lash, R. Steve Wagner

**Affiliations:** 1 Graduate School of Biosphere Science, Hiroshima University, Higashi-Hiroshima 7390046, Japan; 2 Primate Behavior and Ecology Program, Central Washington University, Ellensburg WA 98926, USA; E-Mails: Alexander.Pritchard@Rutgers.edu (A.J.P.); alexdl@comcast.net (A.S.D.); 3 Department of Anthropology, Central Washington University, Ellensburg WA 98926, USA; E-Mail: SheeranL@cwu.edu; 4 School of Life Sciences, Anhui Normal University, Wuhu 241000, China; E-Mail: jhli@ahu.edu.cn; 5 School of Resources and Environmental Engineering, Anhui University, Hefei 230601, China; E-Mail: wangxi198307@163.com; 6 Department of Biological Science, Central Washington University, Ellensburg WA 98926, USA; E-Mails: lixing@cwu.edu (L.S.); WagnerS@cwu.edu (R.S.W.); 7 Department of Anthropology, Rutgers University, New Brunswick, NJ 08901, USA

**Keywords:** conservation, ethnoprimatology, human-macaque interactions, macaque tourism, monkey park, park ranger, wildlife management, wildlife tourism

## Abstract

**Simple Summary:**

Conflict between macaques and humans is a commonly reported problem in Asian tourism. However, without understanding how macaques are managed, the establishment of an effective management design is impracticable. This study explored how monkeys were managed and tourists were regulated at the Valley of the Wild Monkeys in Mt. Huangshan, Anhui Province, China, through a field observation. Two teams of park rangers alternated monthly and managed a group of macaques. The results suggested that undesired tourists’ interactions with monkeys were not regularly intervened by park rangers, and park rangers established dominance over the monkeys by using physical threats to manage them.

**Abstract:**

Previous studies have reported the negative impacts of tourism on nonhuman primates (NHPs) and tourists and advocated the improvement of tourism management, yet what constitutes good quality management remains unclear. We explored whether rates of macaque aggression and self-directed behaviors (SDBs) differed under the supervision of two park ranger teams at the Valley of the Wild Monkeys (VWM) in Mt. Huangshan, Anhui Province, China. The two ranger teams provisioned and managed a group of macaques on an alternating monthly basis. Monkey, tourist and ranger behaviors were collected from August 16–September 30, 2012. Macaque aggression and SDB rates did not differ significantly under the management of the two teams. Overall, there was little intervention in tourist-macaque interactions by park rangers, and even when rangers discouraged tourists’ undesirable behaviors, tourist interactions with monkeys persisted. Furthermore, only one or sometimes two park rangers managed monkeys and tourists, and rangers established dominance over the monkeys to control them. In order to effectively manage tourists and monkeys by a single park ranger, we recommend that rangers: (1) prohibit tourists from feeding; (2) move around the viewing platform more frequently; and (3) limit the number of tourists each visiting session.

## 1. Introduction

Monkeys classified in the genus *Macaca* are commonly found in an anthropogenic environment and exploit overlapping resources with humans. Therefore, they inevitably attract the attention of tourists [1,2]. A variety of human-macaque interactions occur at such tourism sites, and these interactions are frequently studied in the field of ethnoprimatology, which considers humans and nonhuman primates (NHPs) as aspects of the environment and attempts to understand behaviors and the ecology of NHPs from a holistic perspective [3]. Taking an ethnoprimatological approach is essential to establish an effective management strategy, particularly in tourism settings intended to conserve NHPs, while conversely placing high human activity pressure on NHPs. Previous ethnoprimatological studies of macaque tourism have primarily focused on interactions between tourists and monkeys. These studies reported increases in macaques’ rates of aggressive behaviors directed toward humans and conspecifics [4,5,6,7,8] and documented potential risk of disease transmission [9,10,11,12]. Some studies also found an increase in monkeys’ rates of self-directed behaviors (SDBs) [13,14], which are indicators of stress levels [15]. Aggression directed toward tourists is particularly likely when site visitors feed monkeys or other wildlife [16]. The frequency and duration of agonistic interactions among Formosan macaques (*M. cyclopis*) at Shou Shan National Park, Taiwan, significantly increased with tourists providing food to the monkeys [17], and Tibetan macaques (*M. thibetana*) at Mt. Emei, China, attacked tourists to obtain food [7]. More than half of Barbary macaque (*M. sylvanus*) bites reported at Upper Rock Nature Reserve, Gibraltar, were provoked by the tourists who fed the monkeys [4]. Although well-habituated macaques can become quite bold around humans, particularly when humans feed them, studies indicate that tourists, not monkeys, initiated the majority of tourist-macaque interactions (e.g., *M. cyclopis* [17]; *M. thibetana* [18]). Collectively, these and other studies are of concern, because they indicate that macaque tourism is harmful to wildlife, ultimately undermining the conservation value of these tourist experiences.

While several studies have explored the impacts of tourism on nonhuman animals, Curtin [19] noted that researchers have disregarded the role tourism personnel may play in managing tourists’ interactions with wildlife. Their roles can be critical at naturalistic tourism sites, where physical barriers are absent and visitors can directly interact with wildlife [19,20]. Park employees may need to mediate the behaviors of tourists and their proximity to wildlife at such sites [21]. Monkeys at macaque tourism sites are lured (usually with food) by park rangers from surrounding forests to viewing areas [22], and some tourists have high expectations of seeing wildlife [23]. Park rangers are often skilled at locating the wildlife that visitors want to view, and they may additionally protect tourists from aggressive animals. Appropriate mediation of tourist-animal interactions by tourism personnel may reduce some of the negative aspects of tourism. Monkey parks in Japan successfully improved their management of free-ranging macaques by limiting feeding to authorized park staff and strictly prohibiting tourists from feeding monkeys, resulting in a reduction of tourist-macaque conflict [24].

Just as few scholars have studied the role park staff or rangers play in nature-based tourism, little is known about the influence management quality has on wildlife and tourists. Fuentes, Shaw and Cortes [25] compared human-macaque interactions at Padangtegal Monkey Forest in Bali, Indonesia, and the Upper Rock Nature Reserve in Gibraltar. Both sites had signs warning tourists to avoid interactions with macaques. The majority of long-tailed macaque (*M. fascicularis*) groups in Padangtegal were provisioned at shrine or temple locations, but staff ignored tourist-macaque interactions unless the monkeys became aggressive. The number of tourists bitten by macaques and monkey aggression rates at Padangtegal were reportedly higher than what were observed in Gibraltar, where a government agency, the Gibraltar Ornithological and Natural History Society (GONHS), managed Barbary macaques. These differing results, in addition to species-specific behavior patterns, might be partly attributable to the varied demography of tourist groups. For example, macaques in Padangtegal were more frequently visited by humans, whereas the monkeys at Gibraltar encountered tourists less often. Furthermore, some differences appeared to be a consequence of management practices. For example, temple staff at Padangtegal ignored tourist-macaque interactions, while GONHS staff more strictly enforced the rules. However, Fuentes and colleagues [25] did not compare how the different management styles used at the two sites might have impacted tourist and macaque behaviors. To date, no macaque tourism study has been conducted on the potential impact that the management aspect has on tourists’ and/or monkeys’ behaviors.

Co-author Lori K. Sheeran anecdotally noted in previous years that park rangers varied in their management styles and that monkeys and tourists were more responsive to some rangers. In this study, we compared park rangers’ management practices and their potential effects on tourists and monkeys at the VWM, Mt. Huangshan, China. We tested the effect of tourists’ behaviors on monkeys with two predictions: (1) the intensity levels of tourist behaviors would positively correlate with monkey aggression and SDB rates; and (2) under the supervision of rangers who regulated tourist behaviors, lower levels of macaque aggression and SDBs would be observed.

## 2. Methods

### 2.1. Study Site and Subjects

The study was conducted August 16 to September 30, 2012 at VWM (30°07'09''N, 118°09'41''E), Anhui Province, China. The study site is adjacent to Mt. Huangshan, a UNESCO World Heritage Site. At VWM, Tibetan macaques have been the subjects of tourism since 1994 [26]. Tourists ascend stairs to a viewing platform from which they can observe the macaques. Typically, 3–4 tourist groups visit one of two habituated monkey groups, Yulingkeng A1 (YA1) and Yulingkeng A2 (YA2), each day during the summer months [26]. Park rangers scatter corn widely in the provisioning area to lure the monkeys, so that tourists can view them more easily. Tourists pay 40 CNY (7 USD) and find their own way to the viewing platform by following signs and paved trails, or they can pay 60 CNY (10 USD) to have a guide accompany them to the viewing platform.

VWM is run by a private company that owns several nature parks in China. Two pairs of park rangers work for VWM and rotate on the 16th of each month between monitoring YA1 and YA2. Their required tasks include managing the monkeys and regulating tourist’s interactions with the monkeys. We focused our study on group YA1, because staff had difficulty controlling the movements of group YA2 at the time of our project, so more tourists were being directed to the YA1 group. Members of YA1 have been studied since 1986, and all adult monkey identities were known. In 2012, the YA1 group consisted of four adult males, eight adult females, three sub-adults, 12 juveniles and five infants (one was born during the data collection period). Tourist(s) and park ranger(s) who were present on the YA1 viewing platform comprised our research subjects, in addition to all YA1 adult monkeys on the viewing platform and/or in the provisioning areas adjacent to the viewing platform.

### 2.2. Procedures

Data collectors achieved ≥90% inter-observer reliability on monkey identification and behaviors August 6 to 15, 2012 before formal data collection began. All of our data (about monkeys, tourists, rangers) were collected from the viewing platform. Three observers collected data on macaque aggression and SDBs using Berman and colleagues’ [27] published ethogram. Aggression directed toward conspecifics and humans were combined. Co-authors Alexander J. Pritchard and Alexander S. DuVall-Lash conducted randomized, 5-min focal samples with two alterations to the original ethogram: short and long lunges were combined, and slow grab was categorized into grab. Behaviors were recorded as occurring in the presence or absence of tourists. Co-author Lori K. Sheeran conducted 2-min scan samples on monkeys and recorded all-occurrences of aggression and SDBs, the number of monkeys present in the provisioning area or on the viewing platform and the duration of each tourist session (see below).

A tourist session consisted of a minimum of one tourist on the viewing platform and one monkey present in the provisioning area or on the platform for ≥2 min. Co-author Rie Usui recorded tourist behaviors using a SONY digital video camera (Handycam DCR-SX45) and an outdoor remote camera. The remote camera was mounted in the monkeys’ feeding area on the highest level of the viewing platform. It was operated by joystick from the Anhui University Field Laboratory approximately 1 km away from viewing platform. The camera was adjusted daily to a set position where the viewing platform was visible. Data recorded with the remote camera were collected on the main computer system in the laboratory and downloaded each day. The remote camera was used from August 29–September 8, when the system broke. The Handycam DCR-SX45 was used on the viewing platform and positioned to capture tourist behaviors.

Co-author Rie Usui coded all video data about tourist behaviors adapted from an ethogram ([18,28]; but we excluded barbed-wire shake due to the removal of barbed wire), which were classified into high, medium and low intensity levels for analysis. High-intensity tourist behaviors occurred ≤arm’s length with monkeys or >arm’s length with monkeys if tourists held (an) object(s) or food handout(s) (e.g., from the ethogram, touch, kick, show rock, throw corn). Medium-intensity tourist behaviors occurred >arm’s length and ≤2 m in proximity with monkeys without objects or food handouts (e.g., from the ethogram, dangle body parts, point, wave). Low-intensity tourist behaviors occurred >2 m proximity away from monkeys and involved no object or food handout. Co-author Rie Usui and a research assistant independently watched the collected video clips, which were 60 min in total length. They included a wide range of tourist behaviors with ≥90% inter-rater reliability.

Ruesto *et al.* [28] found that decibel readings positively correlated with rates of monkey aggression at VWM, so we measured noise as a potential aspect of management. Co-authors Alexander S. DuVall-Lash and Alexander J. Pritchard recorded decibel levels at the beginning and end of each monkey focal sample using an Extech 407730 40-to-130-Decibel Digital Sound Level Meter. To obtain consistent measurements, we recorded decibel levels from the same location on the viewing platform each time using the same technique. For each tourist session, co-author Rie Usui noted the presence or absence of a tour guide.

**Table 1:** Definitions of park ranger behaviors.

Tourist-Directed	Definition
*Intervene*	Park ranger physically and/or vocally interferes when tourist-macaque interactions occur.
*Restrict*	Restrict tourists within certain areas of the viewing platform.
*Escort*	Ascend/descend the stairs with tourists.
*Confiscate*	Confiscate food tourists brought to the viewing platform.
*Give tour*	Lecture tourists using a microphone.
*Offer*	Offer tourists corn and/or cigarettes.
*Object threat*	Use of objects to threaten (e.g., show/throw rock).
*Vocal threat*	Yell at and/or make a sound toward the monkeys.
*Gestural threat*	Use body parts to threaten (e.g., approach, point).
*Monkey threat*	Mimic a monkey threat (e.g., open mouth, stare).
*Feed non-provisioned food*	(Hand) feed/show non-provisioned food.
*Contact*	Contact with monkeys physically or non-physically (e.g., touch, kick, sneeze, spit).

Co-author Rie Usui recorded all-occurrences of park rangers’ behaviors from an ethogram designed for this study (Table 1). Individual ranger behaviors were aggregated and reported as occurring by Team A and Team B. We combined these data to protect rangers’ identities and because the rangers on each team had the same management routines (e.g., provisioning style, regulation of tourist-macaque interactions). Additionally, the two rangers on one team were often present at the provisioning site during the same hours and simultaneously managed the site, so their individual influences were mostly inseparable. A “provisioning session” occurred when an entire bowl of corn (approximately 2 kg) was distributed throughout the provisioning area by park rangers.

Central Washington University’s Institutional Animal Care and Use (A011204) and Human Subjects (H12103) Committees reviewed and approved our study methods prior to data collection. The latter stipulated the protection of individual ranger’s identities, as the behaviors we recorded could potentially impact ranger employment or censure.

### 2.3. Data Analysis

Unless otherwise stated, the significance level was set at ≤0.05 for all statistical tests. We tested the assumption that the two teams had different management practices by comparing each team’s mean number of daily provisioning and mean latencies of provisioning upon tourists’ arrival at the viewing platform using Mann–Whitney *U*-tests. We compared the decibel levels of the two management teams using independent samples *t*-tests and descriptively reported occurrences of park rangers’ intervention in tourists’ interactions with monkeys. We then calculated rates of monkey and tourist behaviors per individual per minute or hour to compare the impact that the two ranger teams had on monkeys and tourists.

Our analysis consists of two parts. First, we checked for the effect of tourist behaviors on rates of monkey aggression and SDBs, as monkeys might have been affected by different groups of tourists in each session. We ran Pearson’s correlations to test whether the number of tourists and rates of high-, medium- and low-intensity tourist behaviors covaried with rates of monkey aggression and SDBs. Alpha was adjusted using Bonferroni correction for multiple comparisons, resulting in a value of 0.0125 (0.05/4 variables). Second, we tested to determine whether monkeys’ rates of aggression and SDBs differed between the two pairs of rangers using a Mann–Whitney *U*-test. We used the Mann–Whitney *U*-tests to compare the rates of tourist behaviors of high, medium and low intensities between ranger Teams A and B and between guided and self-guided tour groups.

## 3. Results

### 3.1. Rangers

We observed Team A for 865 min over 20 days and Team B for 441 min over 11 days. Although mean numbers of daily provisioning (Mann–Whitney *U*-test; *U* = 108, *z* = −1.56, *p* = 0.119) were not significantly different between the two park ranger teams, the mean latency of Team A’s provisioning (*M* = 16.9 min, *SD* = 2.18 min) upon tourists’ arrival on the viewing platform was significantly longer than Team B (*M* = 11.1 min, *SD* = 3.98 min) (Mann–Whitney *U*-test; *U* = 676, *z* = −1.975, *p* < 0.05). In addition, the noise level when Team A (tourist *M* = 66.8, *SD* = 3.59; no tourist *M* = 63.4, *SD* = 1.86) was on duty was significantly higher than Team B (tourist *M* = 63.2, *SD* = 3.49; no tourist *M* = 60.1, *SD* = 1.93), both during no tourist and tourist sessions (independent *t*-tests; no tourist *t* (1,061) = 25.6, *p* < 0.001; tourist *t* (1,046) = 14.7, *p* < 0.001). Team B rangers fed a handful or several kernels of corn approximately every 20 min (22 observations/441 min) and fed other types of food, such as fruit, to the monkeys every day (11 observations/11 days). Such interactions with monkeys were not observed in Team A.

The rates of rangers’ behaviors directed toward tourists and monkeys are reported in Table 2. Rangers usually asked tourists to stay on the viewing platform during tourist sessions and sometimes escorted tourists up or down the stairs, particularly when monkeys were near the path the tourists were using. Ranger Team A confiscated the food that tourists brought to the viewing platform and on one occasion gave an informal lecture to the tourists using a microphone. However, both teams engaged in behaviors that could potentially encourage tourists to violate the park’s non-smoking and non-feeding policy (Team A, two observations/20 days; Team B, 10 observations/11 days).

**Table 2:** Hourly rates of park ranger behaviors for ranger Teams A and B. Rates were adjusted for each ranger.

**Tourist-Directed**	**Team A** rate of behavior (number/hours recorded)	**Team B** rate of behavior (number/hours recorded)
*Intervene*	0.63 (9/14.4)	0.00 (0/7.35)
*Restrict*	0.56 (8/14.4)	0.41 (3/14.4)
*Escort*	0.49 (7/14.4)	0.14 (1/7.35)
*Confiscate*	0.07 (1/14.4)	0.00 (0/7.35)
*Give tour*	0.07 (1/14.4)	0.00 (0/7.35)
*Offer*	0.14 (2/14.4)	0.82 (6/7.35)
**Monkey-directed**	**Team A** rate of behavior (number/hours recorded)	**Team B** rate of behavior (number/hours recorded)
*Object threat*	0.90 (13/14.4)	0.07 (0.5 */7.35)
*Vocal threat*	0.21 (3/14.4)	1.02 (7.5 */7.35)
*Gestural threat*	0.69 (10/14.4)	1.77 (13/7.35)
*Monkey threat*	0.07 (1/14.4)	0.41 (3/7.35)
*Feed non-provisioned food*	0.00 (0/14.4)	3.47 (25.5 */7.35)
*Contact*	0.14 (2/14.4)	0.00 (0/7.35)

Note: * Indicates cases where two rangers were present simultaneously, and the summation of ranger behaviors resulted in an odd number. Hence, division of these numbers by two yielded decimal numbers.

Park rangers collectively intervened to protect tourists and/or researchers from monkeys nine times across seven tourist sessions (Table 3). Team A intervened during eight out of 33 total sessions (24.2%), where we observed high-intensity tourist interactions. All incidents of intervention entailed monkeys being on the viewing platform and within an arm’s length from tourists. High-intensity tourist interactions occurred during 19 sessions when Team B was on duty, but no intervention was observed.

To regulate monkey behaviors, the rangers in both teams used vocal commands, gestures (e.g., finger point) and objects (e.g., show/throw rocks) (Table 2). Occasionally, some of the rangers had direct physical contact with monkeys. One team, in particular, provoked aggression from the monkeys with their frequent interactions with them every 22 min (20 times/441 min). While resting during the day, the rangers were occasionally observed to position a bowl of corn under their seats, presumably to keep the monkeys proximate when tourists were absent.

**Table 3:** Contexts of park ranger Team A interventions in tourist-monkey interactions. Team B was not observed to intervene in tourist-monkey interactions.

Context	Intensity
A few monkeys climbed onto the viewing platform where >10 tourists gathered. A ranger approached and showed a rock to the monkeys.	High
A mother and her son were trapped as a couple of monkeys on the viewing platform blocked their path. A ranger approached them, which resulted in the monkeys’ retreat from the viewing platform.	High
A tourist left a plastic bag on the viewing platform. A juvenile monkey searched in the bag. A ranger yelled at the monkey. The monkey retreated.	High
Two researchers were trapped as several monkeys were on the viewing platform and blocked their way. A ranger chased the monkeys away.	High
A tourist fed monkeys. The beta male (Zilong ) came to the viewing platform and approached the tourist. A ranger warned the tourist, but feeding persisted. A few juveniles and an adult female (Huahong ) came into close proximity (within an arm’s length). The ranger warned again. The tourist retreated.	High
Several tourists hand-fed an adult female (Yezhen ) with corn and non-provisioned food. The alpha male (Tougui) and another adult female (Huahui) came to the viewing platform and begged the tourists. A ranger approached and chased the monkeys away.	High
A tourist stood right next to an adult female monkey (Yehong) sitting on the platform railing for a photograph opportunity. A ranger approached toward the area of the interaction. Yehong hid behind the wooden board that covered the railings, but did not retreat.	High
A tourist fed non-provisioned food to an adult female monkey (Yehong). Another tourist attempted to touch Yehong. Yehong showed aggression toward the tourist. A ranger chased Yehong away, but she came back soon after the ranger was gone.	High
A little boy climbed on the platform rail, but stayed within the viewing platform and monkeys were >2 m away.	Low

### 3.2. Monkeys

For the first part of our main analysis, overall rates of monkeys’ aggression and SDBs were neither significantly correlated with the number of tourists present on the viewing platform (aggression *r* (82) = −0.040, *p* = 0.723; SDBs *r* (82) = −0.056, *p* = 0.615), nor with the intensity of the tourists’ behaviors while they were on the viewing platform (high-intensity: aggression *r* (82) = 0.047, *p* = 0.672; SDBs *r* (82) = −0.058, *p* = 0.605; medium-intensity: aggression *r* (82) = 0.119, *p* = 0.288; SDBs *r* (82) = 0.037, *p* = 0.743; low-intensity: aggression *r* (82) = 0.079, *p* = 0.481; SDBs *r* (82) = −0.008, *p* = 0.945). For the second part of our main analysis, monkey’s overall rates of SDBs and aggression did not differ significantly between ranger teams A and B (Mann–Whitney *U*-test; aggression *U* = 658, *z* = −0.837, *p* = 0.403; SDBs *U* = 682, *z* = −0.602, *p* = 0.547). Thus, we found no relationship between management teams, tourists’ behaviors or numbers of tourists and the rates of the monkeys’ aggression or stress-related behaviors.

We observed the alpha male (Tougui) self-biting (*n* = 9), sometimes accompanied by spinning. Wounds were visible on the inside of his elbows and upper legs, where the biting occurred. One ranger-alpha male interaction involving non-provisioning food and the ranger’s monkey-directed threats were immediately followed by the alpha male engaging in a sequence of multiple self-bites and aggression directed toward the ranger.

### 3.3. Tourists

We observed a total of 82 tourist sessions. Tourist sessions lasted on average 15.9 min (*SD* = 8.70 min) with an average of 25 tourists per session (*SD* = 19.2 tourists). In addition to all of the behaviors from the tourist behavioral ethogram [18,28], we saw tourists kick monkeys (*n* = 1), touch or attempt to touch monkeys (*n* = 2), play a birdcall to the monkeys (*n* = 1) and a young child urinated on the viewing platform (*n* = 1). Tourists sometimes brought food, including fruits, steamed buns, boiled eggs and packaged snacks (*n* = 225). We aggregated tourist behaviors according to intensity and noted average rates of 2.76 behaviors/hour/tourist (*SD* = 3.72) in the low-intensity category, 5.16 behaviors/hour/tourist (*SD* = 6.6) in the medium-intensity category and 2.52 behaviors/hour/tourist (*SD* = 7.2) in the high-intensity category. High-intensity tourist interactions were observed during 52 out of 82 tourist sessions and occurred at a significantly higher rate under management of Team B (Mann–Whitney *U*-test; *U* = 478, *z* = −2.67, *p* = <0.05). A tour guide was present for 52 sessions (63.4%), but rates of tourist behavior (for all intensity levels) were not significantly different between guided and non-guided tourist groups (Mann–Whitney *U*-test: High *U* = 667, *z* = −1.12, *p* = 0.263; Medium *U* = 614, *z* = −1.60, *p* = 0.11; Low *U* = 744, *z* = −0.35, *p* = 0.725). We did not observe any physical injuries to monkeys or to tourists.

## 4. Discussion

The goal of this study was to determine whether interventions of undesired tourist-macaque interactions by park rangers would be an effective method in lowering rates of monkey aggression and SDBs. Our results showed that the park rangers often worked separately to manage the site, seldom regulated tourist-macaque interactions at VWM and that such interactions sometimes persisted even when interventions occurred. In the following discussion, the details of our findings with respect to park rangers, tourists and macaques are reviewed.

### 4.1. Rangers

Park rangers often individually managed a group of monkeys and tourists at VWM, a phenomenon which was also commonly seen at monkey parks in Japan, because in both locations, financial constraints prohibit employing more rangers [22]. Park rangers at both locations, therefore, are tasked with both controlling the movement of the monkeys and overseeing tourist-macaque interactions [22].

We found that park rangers rarely and minimally regulated tourist-macaque interactions, which suggests that the majority of tourists interacted with the monkeys. Qingming *et al.* [2] interviewed park employees at Nanwan Monkey Island in Hainan, China, and reported that they consider permission of tourist-macaque interactions appropriate. Therefore, the park rangers at VWM may have a similar belief.

Ranger Team A most often used objects as a means of disincentive to control monkeys, which were followed by gestural threats, whereas ranger Team B most frequently used gestural threats followed by vocal threats. The rangers told co-author Xi Wang that they established their dominance over monkeys through the use of threats directed toward the monkeys. Overall, gestural threats were most commonly used, and in many cases, a look or a word from a ranger would cause an aggressive monkey to retreat. Although the platform was set up to divide the space shared between tourists and monkeys, the monkeys often climbed up on the platform in an attempt to solicit food from tourists. In such cases, some rangers occasionally used more severe punishment to deter monkeys from doing so.

VWM rangers appeared to attempt to keep the monkeys within their viewing range. Because the monkeys are free-living, they tended to travel back up into the mountains after provisioning and tourist sessions. One ranger blocked the monkeys’ travel path to one side of the mountain after provisioning by sitting on the extension of the platform where the monkeys needed to pass in order to go up into the woods. This side of the mountain is steep, and it is difficult to find the monkeys once they disappear. The ranger explained to co-author Xi Wang that he wanted to block the pathway to this mountainside. Other rangers continuously fed corn or showed non-provisioning food to the monkeys to lure them back when they began to move away from the viewing area. Sometimes, a ranger would put a bowl of corn on the platform so that the food was continually visible to the monkeys. This was intended to ensure that the monkeys remained nearby. Toward the end of our study, the monkeys appeared to spend more time in the woods adjacent to the viewing platform, perhaps due to an increased abundance of naturally occurring foods during autumn. Rangers’ food calls to the monkeys were sometimes accompanied by their dangling apparently more desirable foods, such as fruit, into the provisioning area, particularly when the monkeys stopped coming down from the mountains to feed on corn. This strategy worked for some monkeys; however, the others remained stationary.

Implementing the above management strategies is likely due to the limitation in the number of park rangers at work relative to the size of the monkey group and the number of tourists. At VWM, one or sometimes two park rangers were on duty simultaneously. Thus, controlling the movement of monkeys and regulating tourists’ behaviors were entirely the responsibility of a single park ranger. Although it seems inappropriate that the park rangers physically threaten monkeys, letting the monkeys know that the park rangers are dominant may, in turn, create easier conditions in controlling the monkeys when tourists were present. The same method is practiced at monkey parks in Japan, where park rangers physically punish aggressive monkeys based on the belief that the park rangers must place themselves at the top of macaque social hierarchy in order to effectively manage the monkey troops [22].

### 4.2. Monkeys

We found no difference in overall rates of monkey aggression and SDBs between the two ranger teams, perhaps because the rangers practice minimal regulation of tourist-macaque interactions.

The observation of self-biting by the alpha male raises concerns about the welfare of the monkey, because in captive nonhuman primates, self-biting is difficult to eradicate and often requires treatment, such as medication [29].

Previous research conducted at VWM reported associations between rates of macaque aggression and SDBs with various features of tourist groups [14,18,28]. Like Ruesto *et al.* [28], we did not find a significant correlation between macaque aggression rate and tourist number. Matheson and colleagues [14] found a positive correlation between rates of SDBs and tourist numbers at particular viewing areas where the monkeys were adjacent to and flanked by tourists on two sides, which suggests that monkeys are not equally stressed throughout the provisioning area by the tourists’ presence. In contrast to Matheson *et al.* [14], our data did not show significant correlations between rates of SDBs and tourist numbers. This may be because our macaque behavioral data were collected from the entire provisioning area. McCarthy *et al.* [18] reported that tourists’ finger pointing provoked the most aggression from macaques, while we found no significant correlation between tourist behaviors and rates of monkey aggression. This discrepancy may result from the categorization in the present study of various tourist behaviors based on three levels of intensity.

Although neither rates of tourist behaviors nor tourist numbers had significant effects on monkeys’ aggression and SDB rates, noise levels seemed to affect both types of behavior. DuVall-Lash [30] compared rates of monkey aggression and SDBs across three noise levels (high, medium and low) that were defined according to deviations from the mean. He categorized all decibel measurements below −1 standard deviation as low arousal, those from −1 to +1 standard deviations as medium arousal and those above +1 standard deviation as high arousal (low arousal < 60.5 db, 60.5 ≤ medium arousal ≤ 69.1 db and high arousal > 69.1 db). The noise levels of the two ranger teams were significantly different, both when tourists were present and absent, but rates of monkey aggression and SDBs were not significantly different between the two ranger teams. This appeared to contradict DuVall-Lash’s [30] finding that different noise levels significantly affected monkey aggression and SDB rates. One possible explanation for this discrepancy is that the decibel levels of the two teams were not different enough to clearly show significant differences in rates of monkey aggression and SDBs.

### 4.3. Tourists

We found a significantly higher rate of high-intensity tourist behaviors under management of Team B compared to Team A. One ranger in Team B offered corn to tourists for them to feed the monkeys, which likely increased the rates of high-intensity tourist behaviors.

Our data showed that the management style of VWM was similar to the temple staff’s management style in Bali, where interventions only occurred when tourists provoked an aggressive interaction from the monkeys [25]. According to Qingming *et al.* [2], Chinese people consider that closer interactions and associations with wild animals reflect the harmonious state of a human relationship with animals. In addition, the Chinese believe that they have little influence in affecting wild animals [2].

The rangers intervened in tourist-macaque interactions with the alpha and/or beta males or when several monkeys gathered on the viewing platform in close proximity to tourists. Knight [22] saw a similar management style among smaller monkey parks in Japan, where the rangers’ primary focus was managing monkeys rather than overseeing tourists. Safety instructions or warnings were rarely given verbally to tourists at VWM, but this may be partly because tourists rarely listen (see also [2]). At VWM, tourist interactions with the monkeys persisted even when the rangers discouraged the tourists’ undesirable behaviors. Some rangers had a practice of confiscating foods that tourists brought for the monkeys, which, after confiscating, the rangers then used to feed the monkeys. This may have been in an attempt to terminate tourist interactions with the monkeys and a strategy to keep the monkeys off the viewing platform, since the ranger, rather than the tourist, controlled when and where the handout would occur.

Rangers were tasked with maintaining visual contact with the monkeys and luring them down to the provisioning area for the tourists to view. Periodically, the rangers had to leave the platform to search for monkeys in the mountains. Tourists sometimes arrived, while the rangers were absent from the viewing platform, which left no one on the platform to regulate tourist behaviors and mediate macaque and tourist interactions if the monkeys arrived at the provisioning area and/or platform before the rangers. Some tourists would enter the provisioning area to bathe their hands and faces in the stream, which monkeys were observed to drink from and swim in. Tourists may have been exposed to monkey-borne pathogens. Previous studies found that some members of YA1 tested positive for herpes B virus, hepatitis A virus, simian foamy virus, simian pox virus, simian retrovirus or simian T-cell lymphotropic virus-1 [9]. Posting signs to keep tourists from entering the provisioning zones could help to regulate such tourist behaviors, but it is likely that ranger presence is the best deterrent of this behavior.

Signs intended to regulate tourists’ behaviors were located on the viewing platform and at the park entrance. At the viewing platform, two rules were posted: “no feeding” and “no smoking”. However, feeding by tourists frequently occurred, which suggests that tourists ignored or did not read the signs at VWM, as has been observed at other macaque tourism sites [2,25]. The non-smoking sign was displayed at a 90° rotation, which may make it less effective in regulating such tourist behavior. In addition, the signs were not at eye-line and were posted above a threshold within the platform, reducing their potential visibility.

The size of tourist groups could limit rangers’ abilities to monitor and regulate monkeys’ and tourists’ behaviors. The largest tourist groups we observed exceeded 100 people, which made the platform over-crowded. Moreover, tourists were sometimes barred from using the lowest level of the platform, typically because park rangers attempted to keep the tourists from being widely spread out on the platforms. When one level was closed, even a medium-sized tourist group crowded the platform, and the rangers could not move freely among the people. Limiting the number of tourists per visiting session would allow the rangers to better supervise the tourists and the monkeys. Some wildlife parks control the number of visitors entering during peak seasons or times by increasing the entrance fee [21]. The fact that many VWM tourists paid extra for a guided tour might indicate a willingness to pay more for a better wildlife viewing experience.

At VWM, tour guides provided information regarding the monkeys, provisioning and appropriate tourist behaviors. Tourists were required to pay an additional fee to be accompanied by a tour guide (40 CNY for general admission, 60 CNY for a guided tour). Guided tourist sessions occurred 63.4% of the time. Tour guides usually introduced monkeys by name, including their kinship, along with information about the species’ social structure, behaviors and biological features. In addition, tour guides explained why corn was distributed widely across the viewing area. Tour guides gave tourists guidelines for appropriate interactions with the monkeys (e.g., do not stare at the monkeys) and answered tourists’ questions. Although tour guides gave lectures, information given to tourists varied by tour guide, and it was shown to have no effect on the tourists’ behaviors that we studied (see also [2]). It was unclear what, if anything, tourists learned about the monkeys or their conservation, since they rarely appeared to listen to tour guides or rangers.

## 5. Conclusions

Most ethnoprimatological research at macaque tourism sites has been focused on tourist-macaque interactions and advocated the improvement of management. This study included another group of humans (rangers) to macaque tourism studies and explored their influence on management. By examining the interactions of park rangers with tourists and macaques, as well as tourist-macaque interactions, we gained a better understanding of a complex, triadic relationship (macaques, tourists and park rangers) and suggest changes to improve management for the well-being of all three components. At VWM, the park rangers used various methods to manage monkeys and tourists, and often times, only a single ranger was on duty. Such a management style is also common in Japan, where park rangers alternate their work shift and regulate tourist-macaque interactions [22]. However, at VWM, tourists’ interactions with monkeys were rarely intervened by the park rangers, which may reflect the Chinese cultural belief that having closer interactions with wildlife is a harmonious state of human-animal relationship [2]. It is likely that, due to this minimal regulation of the park rangers of tourist-macaque interactions, we did not find a relationship between rangers’ management practices and rates of monkey aggression and SDBs.

There is room for improved management at VWM, where a single park ranger is expected to manage a group of monkeys and tourists. We recommend prohibiting the tourists from feeding the monkeys, as this aspect of management has been reported to improve the potential tourist-macaque conflicts at monkey parks in Japan [22]. Knight [22] reported that after the implementation of such a change, monkeys no longer associated tourists with food. Eliminating food eased the tension between tourists and monkeys. In addition, the rangers’ frequent movements around the viewing platform may reduce how often monkeys enter the viewing platform. The number of tourists on the platform during each visiting session should be limited to prevent over-crowding and increase the efficacy of the rangers’ influence. This could be accomplished by manipulating the admission rates during the peak tourist season or time of day or by imposing limits on the number of visitors that can visit during specified periods of time throughout the day.
